# STAYING WITH A POSITIVE MIND: STEPPING AWAY FROM ACCELERATED AGING

**DOI:** 10.1093/geroni/igae098.2772

**Published:** 2024-12-31

**Authors:** Yuchen Liu, Wenjie Cai, Eve Wittenberg, Dae Hyun Kim, David Bloom, Laura Kubzansky, Benjamin Seligman

**Affiliations:** Harvard T.H. Chan School of Public Health, Boston, Massachusetts, United States; Johns Hopkins University, Baltimore, Maryland, United States; Harvard T.H. Chan School of Public Health, Boston, Massachusetts, United States; Hebrew SeniorLife, Boston, Massachusetts, United States; Harvard T.H. Chan School of Public Health, Boston, Massachusetts, United States; Harvard T. H. Chan School of Public Health, Boston, Massachusetts, United States; VA Greater Los Angeles Healthcare System, Los Angeles, California, United States

## Abstract

Subjective wellbeing (SWB) is a crucial measure of life quality in older adults. Understanding its relationship with frailty may inform strategies to promote healthy aging. We analyzed data for older adults aged ≥ 60 years old from Waves 3 and 4 of the China Health and Retirement Longitudinal Study. SWB was measured based on participants’ self-reported overall satisfaction with life. A frailty index was developed using the deficit accumulation approach. We conducted a cross-sectional Poisson regression to investigate the relationship between SWB and counts of frailty deficits. Additionally, we conducted a longitudinal analysis to determine the three-year relative risk of clinically significant frailty progression or mortality for different levels of SWB. The analyses were adjusted for individual weights, including adjustments for household nonresponse. The cross-sectional analysis included 9,702 individuals. After adjusting for covariates, lower baseline life satisfaction was associated with higher counts of frailty deficits (mean deficit counts ratio [95% confidence interval]: 1.66 [1.54, 1.78] for “not satisfied” and 1.06 [1.02, 1.10] for “somewhat satisfied” relative to the reference “very satisfied”). The longitudinal analysis included 8,599 individuals. Participants who were “not satisfied” with life at baseline were at a greater risk of frailty progression compared with those who were “very satisfied” (risk ratio: 1.16 [1.00, 1.35]). Our study finds that a lower level of SWB is associated with more severe frailty. It is also associated with frailty progression or death. These results emphasize that both psychological wellbeing and physical health are essential components of healthy aging.

